# PubMed, writing and research

**DOI:** 10.4103/0971-3026.57204

**Published:** 2009-11

**Authors:** Bhavin Jankharia

**Affiliations:** Editor-in-Chief, The Indian Journal of Radiology and Imaging, “F,” 1st Floor, Bhaveshwar Vihar, 383, Sardar V P Road, Mumbai - 400 004, India. E-mail: editor@ijri.org

A month or so ago, I received a heartening news. Dr. D K Sahu, who runs Medknow Publications, which publishes the *Indian Journal of Radiology and Imaging* (IJRI) for us, emailed me saying that the IJRI was finally in PubMed. We had been preparing for this for the last three years and it had finally happened. The IJRI would like to sincerely thank Dr. Sahu and his team for the efforts that they have put in for this.

Now that this promise has been kept, we need to move on. The journal needs to scale greater heights and for this to happen, original articles from India that are currently being published in foreign journals, need to be submitted to the IJRI. From our side, we will ensure timely processing of all articles.

The impact of the PubMed inclusion is already evident—the average submission rate had suddenly increased in the last month; unfortunately, the majority of these are still only case reports. It should be understood that more than 80% of all case reports will be rejected immediately on submission.

It would also help if prospective authors could check whether previous, similar articles have been published earlier in the IJRI. This is easy to do. We have backdated pdf files on www.ijri.org, up till 1999. You can go to Google and type your search word, for example, “lipoma arborescens” followed by “site:ijri.org,” that is, type in the Google search bar, “lipoma arborescens site:ijri.org,” without the inverted commas, and you will get a list of articles with those search words. You can also do this directly using the Search option on the homepage of www.ijri.org. This should be done especially for case reports; if a case report on a particular topic has already been published in the IJRI in the last 10 years, it is almost certain that the submitted case report will be rejected.

We are also planning to focus more on tuberculosis and other tropical infections. The majority of articles on tuberculosis come from either the Western world or South Korea, which is quite sad, considering the amount of data that exist in India, which perhaps far exceed that in other countries. Even our earlier review article on musculoskeletal tuberculosis was written by authors from Belgium. We plan to fast-track original articles, pictorial essays and even unusual case reports on this subject.

It is necessary that students and residents learn to write articles. Apart from the fact that they need to follow the “Guidelines for Authors” strictly, it is important that prospective authors learn how to collate their thoughts and write them out coherently and logically. There is after all a method to the writing madness. Most of the articles submitted to us have extremely poor spelling and grammar. Though this is never a factor that affects the acceptance or rejection of an article, it significantly increases the effort that we have to put in, for reading, reviewing and copyediting. By and large, whenever we find an article significantly language-challenged, we will insist on it being rewritten in conjunction with an English teacher or professor.

Eventually though, more original research needs to be conducted in our various teaching hospitals and institutions. This requires an overhauling of our infrastructure as well as committed and motivated teachers and researchers. We hope that this will happen in the years to come, with some impetus and push from the Central Government as wel-l.

